# Ginsenoside Rh1 suppresses Peste des petits ruminants virus replication by inhibiting autophagy to promote interferon responses and restrict inflammatory responses

**DOI:** 10.3389/fvets.2026.1799680

**Published:** 2026-04-23

**Authors:** Qinglu Zhao, Hongmei Chen, Dingcheng Wei, Zhanying Hu, Xueliang Zhu, Rui Zhang

**Affiliations:** 1 Key Laboratory of Animal Medicine of Sichuan Education Department, Southwest Minzu University, Chengdu, Sichuan, China; 2 State Key Laboratory for Animal Disease Control and Prevention, Lanzhou Veterinary Research Institute, Chinese Academy of Agricultural Sciences, Lanzhou, Gansu, China

**Keywords:** antiviral, autophagy, Ginsenoside Rh1, immunomodulatory responses, Peste des petits ruminants virus

## Abstract

Peste des Petits Ruminants (PPR), a highly contagious disease of domestic and wild small ruminants, is characterized by severe morbidity and mortality. PPRV, the causative agent, is a *morbillivirus* in the family *Paramyxoviridae*. The virus poses a significant barrier to sustainable agricultural development in the developing world. Currently, no effective therapeutics agent for PPRV infection is available. Ginsenoside Rh1, a protopanaxadiol ginsenoside, the major pharmacological ingredient in the plants of ginseng, was reported to inhibit the replication of a broad range of human viruses. However, it is unclear whether Ginsenoside Rh1 can act as an antiviral against PPRV infection. Here, we demonstrate that Ginsenoside Rh1 exhibits significant antiviral activity against PPRV in cell culture models. The mechanism of action of Ginsenoside Rh1 against PPRV is mainly attributed to its ability to block PPRV mediated autophagy, thus leading to stimulation of interferon responses and inhibition of inflammatory responses. In summary, our study establishes Ginsenoside Rh1 as a novel antiviral agent effective against PPRV and potentially other related morbilliviruses, sheds light on its mode of action, and reveals a novel autophagy-dependent dual immunomodulatory strategy that may prove essential for combating both current and future viral outbreaks.

## Introduction

Peste des petits ruminants (PPR), also known as ‘goat plague’, ‘kata’, ‘ovine rinderpest’, or ‘stomatitis-pneumoenteritis syndrome’, is an acute and highly contagious viral disease that primarily affects domestic sheep and goats, while also posing a threat to wild small ruminants. The disease is endemic across extensive regions of Africa, the Middle East, and Asia and is increasingly recognized as an emerging threat in new geographical areas (1). PPR is characterized by high morbidity and mortality rates, which can reach up to 90% in naïve populations (2), leading to severe socioeconomic losses and threatening food security and the livelihoods of farmers in affected regions (3). PPR is currently regarded as one of the fastest-spreading and most economically impactful diseases of small ruminants in the developing world—where these animals are integral to agricultural systems and rural development (4). Due to its substantial impact on animal health and sustainable agriculture, PPR has been targeted by the World Organisation for Animal Health (WOAH) and the Food and Agriculture Organization (FAO) for global eradication by 2030 (5).

Peste des petits ruminants virus (PPRV), the etiological agent of PPR, is an enveloped, negative-sense, single-stranded RNA virus classified within the genus *Morbillivirus* of the family *Paramyxoviridae* (subfamily *Paramyxovirinae*, order *Mononegavirales*) (6, 7). This genus encompasses several highly impactful veterinary viral pathogens, such as rinderpest virus (RPV), canine distemper virus (CDV), marine morbilliviruses phocine distemper virus (PDV), dolphin morbillivirus (DMV) and porpoise morbillivirus (PMV), together with the only human measles virus (MV) (8, 9). Notably, the genus continues to expand, with recent characterizations of novel members such as Feline morbillivirus (10) and numerous morbilli-like viruses identified in bats and rodents (11). A hallmark of PPRV pathogenesis, consistent with that observed for other morbilliviruses, is the induction of a severe yet transient state of immunosuppression (12, 13). This condition significantly heightens susceptibility to opportunistic secondary infections, thereby critically influencing disease severity and mortality (12, 14). The underlying immunosuppression results from a combination of direct viral cytopathic effects within lymphoid cells and the deployment of multiple immune-evasive strategies by the virus (13, 15), which collectively impair both innate and adaptive host immune responses. Thus, there is an urgent need to develop safe and effective antiviral agents against PPRV.

At present, the prevention and control of PPR primarily relies on vaccination, with live attenuated vaccines PPRV Nigeria75/1 (lineage II) and PPRV Sungri 96 (lineage IV) being the most widely used and extensively validated options (16–19). However, these vaccines face substantial practical limitations, particularly in endemic regions. As a *paramyxovirus*, PPRV is inherently heat-sensitive (16, 20), necessitating a continuous cold chain for vaccine distribution—a requirement that is difficult to meet in many PPR-affected countries, which are typically located in hot climates with underdeveloped infrastructure and unreliable electricity supplies. In addition, the high production cost of live attenuated vaccines further constrains their accessibility and large-scale deployment. Although sanitary measures such as movement restrictions and stamping-out policies can theoretically help contain outbreaks, their implementation remains economically and logistically challenging in most endemic settings, which are predominantly in developing countries (16). Compounding these challenges is the fact that no antiviral drugs have yet been approved for the treatment of PPRV infection. Therefore, there is a pressing need to develop safe, effective, and thermostable antiviral agents to complement vaccination and enhance PPR control strategies.

Ginseng, derived primarily from *Panax ginseng*, *Panax quinquefolius* L., and related species, is a widely used herbal medicine in humans known for its immunomodulatory properties (21–23). Among its bioactive compounds, Ginsenoside Rh1 has received significant attention (21, 24, 25) and is employed as a quality control marker for *Panax quinquefolius* L., with a stipulated content of no less than 1.0% (26). Ginseng extracts and their metabolites, such as Rh1, Rb2, Rg3, and intestinal transformation products like Compound K (27), are reported to exhibit various pharmacological activities, such as modulating immune responses (25, 27), exhibiting antioxidative and antibacterial effects (28), and showing potential in alleviating conditions like colitis (29) and diabetes (30). Although fermented ginseng extracts and several ginsenosides have demonstrated broad biological effects—from antioxidative to antidiabetic—their direct antiviral potential, particularly against specific viruses, remains underexplored. Importantly, despite the documented immunomodulatory role of Ginsenoside Rh1 (23, 31, 32), its ability to inhibit PPRV infection has not yet been investigated.

The innate immune system detects viral nucleic acids through specific cytosolic sensors (33): retinoic acid-inducible gene I (RIG-I) and melanoma differentiation-associated gene 5 (MDA5) function as principal sensors for viral RNA (15). Upon detection of viral RNA, RIG-I and MDA5 activate by engaging the mitochondrial antiviral signaling protein (MAVS). Subsequently, MAVS recruits and activates TANK-binding kinase 1 (TBK1), which phosphorylates both MAVS itself and the transcription factor interferon regulatory factor 3 (IRF3). Phosphorylated IRF3 dimerizes and translocates to the nucleus, where it drives the expression of type I interferons (IFN-I) (33, 34). Secreted IFN-I signals in an autocrine or paracrine manner by binding to cell surface receptors, thereby activating the JAK/STAT pathway and leading to the induction of hundreds of interferon-stimulated genes (ISGs), collectively establishing an antiviral cellular state (35, 36). In parallel, MAVS-mediated signaling can also activate the IkB kinase (IKK) complex, leading to the phosphorylation and degradation of IkB proteins, thus releasing NF-kB. Subsequently, NF-κB translocates to the nucleus and induces the production of pro-inflammatory cytokines such as interleukin-6 (IL-6) and tumor necrosis factor-*α* (TNF-α), thereby enhancing inflammatory responses during viral infection (37, 38).

Autophagy, an evolutionarily conserved intracellular lysosomal degradation pathway, serves as a critical mechanism for maintaining cellular homeostasis (39, 40). It facilitates the clearance of damaged organelles, misfolded proteins, and invading microorganisms, thereby protecting cells from metabolic stress, nutrient deprivation, and infection (41–43). Morphologically, autophagy is characterized by the formation of a double-membrane vesicle, the autophagosome, which engulfs cytoplasmic cargo and delivers it to lysosomes for degradation and recycling. This highly regulated process involves three main stages, initiation, elongation, and maturation, orchestrated by over 20 conserved autophagy-related (ATG) proteins that assemble at specific endoplasmic reticulum subdomains upon induction. Key molecular markers for monitoring autophagy include microtubule-associated protein 1 light chain 3 (LC3), which is widely used to track autophagosome formation (44). Beyond its role in cellular quality control, autophagy also plays an integral part in innate and adaptive immunity, participating in host defense against diverse intracellular pathogens such as bacteria, viruses, and protozoa (45, 46). An increasing amount of evidences have shown that the autophagy process can be exploited by PPRV to facilitate its replication (15, 47–49). However, the effects of Ginsenoside Rh1 on morbilliviruses replication and the association between Ginsenoside Rh1 and autophagy have not yet been investigated.

The objective of this study was to assess the impact of Ginsenoside Rh1 on PPRV replication and to investigate its mechanism of action *in vitro*. The resulting data uncover key molecular pathways modulated by Ginsenoside Rh1, which could contribute to the design of novel interventions for controlling PPRV and potentially related viruses like MV.

## Materials and methods

### Cell culture and virus propagation

African green monkey kidney (Vero) cells (ATCC, CCL-81™) were cultured in Dulbecco’s modified Eagle’s medium (DMEM, Gibco) supplemented with 10% heat-inactivated fetal bovine serum (FBS, Gibco) and penicillin–streptomycin solution (100 U/mL and 100 μg/mL, respectively). Caprine endometrial epithelial (EEC) cells were kindly provided by Prof. Yongxi Dou (Lanzhou Veterinary Research Institute, Chinese Academy of Agricultural Sciences, Gansu, China) and cultured in Dulbecco’s minimal essential medium/F-12 Ham’s medium (DMEM/F12) supplemented with 10% fetal bovine serum (FBS, Gibco), 100 IU/mL penicillin, and 10 μg/mL streptomycin. These cells were cultured as monolayers in cell culture flasks or dishes at 37 °C in a humidified atmosphere of 5% CO_2_ in air.

The PPRV attenuated vaccine strain Nigeria 75/1 was obtained from our laboratory’s culture collection. The viral stock was generated by infecting monolayers of Vero cells. PPRV was inoculated into Vero cells and cultured in DMEM supplemented with 2% FBS at 37 °C with 5% CO_2_ for 5 days until a obvious cytopathic effect (CPE) was observed in approximately 80% of the cells. The virus-containing media were collected, and the cells were lysed by three freeze–thaw cycles.

Virus titers were determined by inoculating cell monolayers in 96-well plates with 10-fold serial dilutions of viral stock, followed by incubation at 37 °C for 5–7 days. The 50% tissue culture infective dose (TCID_50_)/mL was calculated using the Reed and Muench method. The multiplicity of infection (MOI) for each experiment was based on the calculated titer for the respective cell line.

### Antibodies and reagents

For Western blotting, the following antibodies were used: Rabbit anti-GAPDH (CST, 5174), rabbit anti-LC3B (CST, 2775), rabbit anti-ATG16L1 (CST, 8089), rabbit anti-Phospho-IRF3 (CST, 29047), rabbit anti-IRF3 (CST, 11904), rabbit anti-Phospho-TBK1 (CST, 5483), rabbit anti-TBK1 (CST, 3504), rabbit anti-Phospho-NF-kB p65 (CST, 3033), and rabbit anti-NF-kB p65 (CST, 8242). Mouse monoclonal antibodies against the N protein of PPRV were obtained from the Lanzhou Veterinary Research Institute, Chinese Academy of Agricultural Sciences. Anti-rabbit IgG, HRP-linked antibody (CST, 7074) and Anti-mouse IgG, HRP-linked antibody (CST, 7076). Ginsenoside Rh1 (HY-N0604) and Rapamycin (HY-10219) were purchased from MCE. Lipofectamine 2000 (11668019) was purchased from Thermo Fisher Scientific.

### Cytotoxicity assay

Cytotoxicity was evaluated using a Cell Counting Kit-8 (CCK-8) assay. Briefly, the cells were seeded in a 96-well plate and cultured for 24 h. Subsequently, the culture medium was replaced with 100 μL of fresh medium containing Ginsenoside Rh1 (MCE, HY-N0604) at various final concentrations (0, 10, 20, 30, 40, and 50 μM). After 48 h incubation at 37 °C under 5% CO_2_, 10 μL of CCK-8 solution was added to each well, followed by a further 2 h incubation at 37 °C. The optical density (OD) at 450 nm was then measured for each set of quadruplicate wells using an ELISA microplate reader (PerkinElmer, VICTOR Nivo™, United States).

### Drug treatment

The cells were seeded into new 6-well plates. At the time of seeding, cells were exposed to varying doses of Ginsenoside Rh1 (0, 10, 20, 30, 40, or 50 μM) for 24 h prior to viral challenge. On the day of infection, cells were infected with PPRV (MOI = 1). The virus inoculum was removed after a 1 h adsorption period at 37 °C under 5% CO_2,_ and the cells were washed to remove unbound viral particles. Subsequently, the cells were cultured in fresh DMEM containing 2% FBS and continuously supplemented with the corresponding concentration of Ginsenoside Rh1 (0, 10, 20, 30, 40, or 50 μM). At 48 h post-infection, cells and corresponding culture supernatants were harvested to determine the dose with the most significant effect on PPRV replication.

The cells were either pretreated with 50 μM Ginsenoside Rh1 for 24 h before infection, 1 h during invasion, 48 h post infection, or both before and after infection, respectively. On the day of infection, cells were infected with PPRV (1 MOI) for 1 h at 37 °C. Both cells and corresponding culture supernatants were harvested at 48 h post-infection to determine on which stage in PPRV life cycle Ginsenoside Rh1 acts.

### Western blotting

The cells, either treated with 50 μM Ginsenoside Rh1 or left untreated, were harvested at 48 hpi. After washing with cold PBS, cells were lysed in RIPA buffer supplemented with protease and phosphatase inhibitors. Lysates were centrifuged to clarify, and protein concentrations were quantified using a BCA assay. Equal amounts of protein were denatured, separated by SDS-PAGE (10% gel), and transferred to PVDF membranes. Following blocking with 5% non-fat milk, membranes were probed overnight at 4 °C with primary antibodies. After washing, incubation with HRP-conjugated secondary antibodies was performed at room temperature for 1 h. Immunoreactive bands were visualized using an ECL substrate. GAPDH was used as a loading control. Images were acquired using a digital imaging system.

### Quantitative real-time PCR

At 48 hpi, total RNA was isolated from cells treated with or without 50 μM Ginsenoside Rh1 using the RNeasy Plus Universal Mini Kit (Qiagen, 73,404). First-strand cDNA was synthesized from the RNA using Maxima H Minus cDNA synthesis master mix (Thermo Scientific, M1682). Quantitative real-time PCR (qPCR) was then performed using PowerUp SYBR Green Master Mix (Applied Biosystems, 1,801,040), with cycling conditions set as per the manufacturer’s protocol. The live-attenuated PPRV Nigeria75/1 vaccine strain served as a positive control. The primers used were as follows: PPRV N forward:

5’-AGAGTTCAATATGTTRTTAGCCTCCAT-3′;

PPRV N reverse: 5’-TTCCCCARTCACTCTYCTTTGT-3’.

GAPDH forward (Vero): 5’-CGAGATCCCTCCAAAATCAA-3’.

GAPDH reverse(Vero): 5’-TGACGATCTTGAGGCTGTTG-3′.

ATG16 forward (EEC): 5’-GGACATGATGGTTCGTGGAA-3′.

ATG16 reverse (EEC): 5’-GGTCAATCACCAACTGGGCTA-3’.

IFN-*β* forward (EEC): 5’-TGCCTCCTCCAGATGGTTCT-3’.

IFN-β reverse (EEC): 5’-TGACCAATACGGCATCTTCC-3’.

ISG15 forward (EEC): 5’-GAAGCAGTTCATCGCCCAGA-3’.

ISG15 reverse (EEC): 5’-ACCTCATAGGAGCTGCTGCG-3’.

IL-6 forward (EEC): 5’-TTCCAATCTGGGTTCAATCA-3’.

IL-6 reverse (EEC): 5’-TTTCCCTCAAACTCGTTCTG-3’.

IL-1β forward (EEC): 5’-ATGCTTCCAATCTGGGTTCA-3’.

IL-1β reverse (EEC): 5’-ATGCTTCCAATCTGGGTTCA-3’.

GAPDH forward (EEC): 5’-CCACGAGAAGTATAACAACACCC-3’.

GAPDH reverse(EEC): 5’-GGTCATAAGTCCCTCCACGAT-3’.

### RNA interference

The siRNAs target *ATG16* gene of EEC cells were synthesized by Sangon Biotech (Shanghai, China). Scrambled siRNA was used as a negative control. The silencing efficiency was measured by quantitative Real-Time PCR. SiRNAs sequences are shown in the following:

siRNA-ATG16 sense: 5’-GCUGAGAAUUAAACACCAAGA-3’.

siRNA-ATG16 antisense: 5’-UUGGUGUUUAAUUCUCAG CUG-3’.

For construction of lentiviral CRISPR-Cas9 vectors, gRNAs are designed using gRNA Designer from Feng Zhang’s lab. Primers are synthesized and cloned into Lenti CRISPR v2 vector by ligation. The target sequence of *ATG16* gene is: 5’-ACAGGAAGCGACATG TCGTC-3′. gRNA sequences are as follows:

gRNA1: 5’-AGATGTGCCGCTTCCAGCGG GGG-3’.

gRNA2: 5’-GCTGCAGAGACAGGCGTTCG AGG-3′.

### Lentivirus packaging and infection

For packaging lentivirus, 1.5 μg psPAX2 packaging plasmid (Addgene, 12,260), 1 μg pMD2. G envelope plasmid (Addgene, 12,259), and 2 μg pLKO.1 plasmid were co-transfected into 4 × 10^6^ Lenti-X 293 T cells using Lipofectamine 2000 transfection reagent. The supernatant was collected at 36 hpi, filtered, and stored at −80 °C. Vero cells were incubated with the viral particles in the presence of 8 μg/mL polybrene (Solarbio, H8761) for 24 h and treated with 5 μg/mL puromycin (Invitrogen, A1113803) for 3 days. The positive monoclonal Vero cells were confirmed by DNA sequencing. The silencing efficiency was measured by immunoblotting analysis.

### Statistical analysis

Data are expressed as means ± standard deviation (SD). The significance of the variability between the different treatment groups was calculated by two-way analysis of variance (ANOVA) using GraphPad Prism software (version 10.0). Differences were considered statistically significant at *p* < 0.05.

## Results

### Ginsenoside Rh1 inhibits PPRV replication

**Figure 1:**
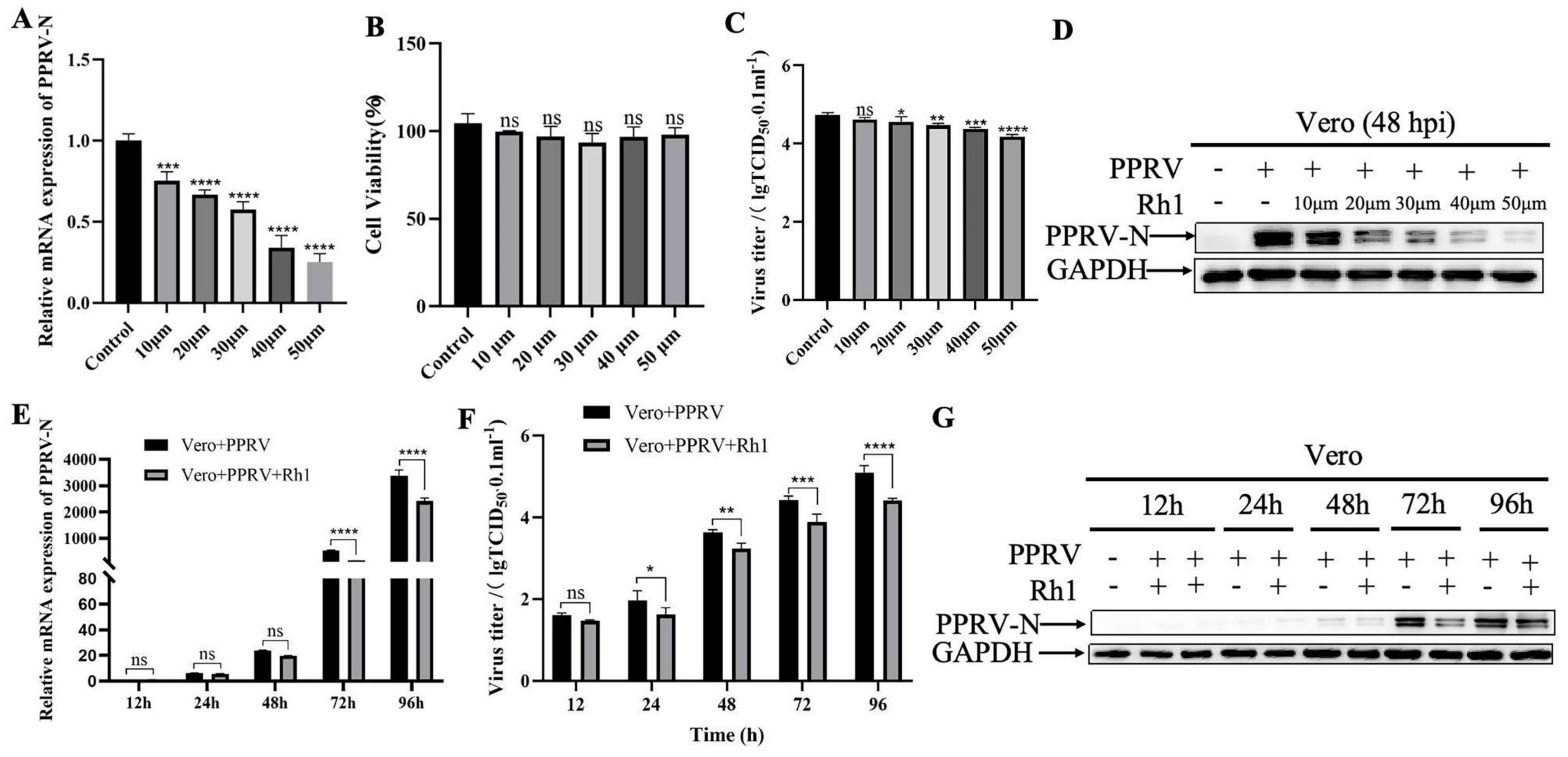
Ginsenoside Rh1 inhibits PPRV replication in a dose-dependent manner. **(A)** PPRV mRNA levels in various concentration Ginsenoside Rh1-treated, PPRV-infected Vero cells (MOI = 1, 48 hpi) were measured by qPCR. The data show the means ± SD; *n* = 3; ***p < 0.001; ****p < 0.0001. **(B)** Cytotoxicity of different concentration of Ginsenoside Rh1. Viability was normalized to non-treated control. The data show the means ± SD; n = 3; ns, no significance. **(C)** Virus titers of various concentration Ginsenoside Rh1-treated, PPRV-infected Vero cells (MOI = 1) were measured by by TCID_50_ (48 hpi). The data show the means ± SD; *n* = 3; **p < 0.01; ***p < 0.001; ****p < 0.0001. **(D)** Western blotting analysis of PPRV N protein in PPRV-infected wild-type and various concentration Ginsenoside Rh1-treated Vero cells (MOI = 1, 48 hpi). GAPDH was used as a loading control. **(E)** PPRV mRNA levels in 50 μM Ginsenoside Rh1-treated, PPRV-infected Vero cells (MOI = 1) were measured by qPCR at different time points. The data show the means ± SD; *n* = 3; ****p < 0.0001. **(F)** Control and (50 μM) Ginsenoside Rh1 pre-treated Vero cells were infected with PPRV (MOI = 1), and virus titers were measured by TCID_50_ at at different time points. The data show the mean ± SD; *n* = 3; *p < 0.05; **p < 0.01; ***p < 0.001; ****p < 0.0001. **(G)** Western blotting analysis of PPRV N protein in PPRV-infected wild-type and Ginsenoside Rh1-treated cells (MOI = 1) at different time points. GAPDH was used as a loading control.

**Figure 2:**
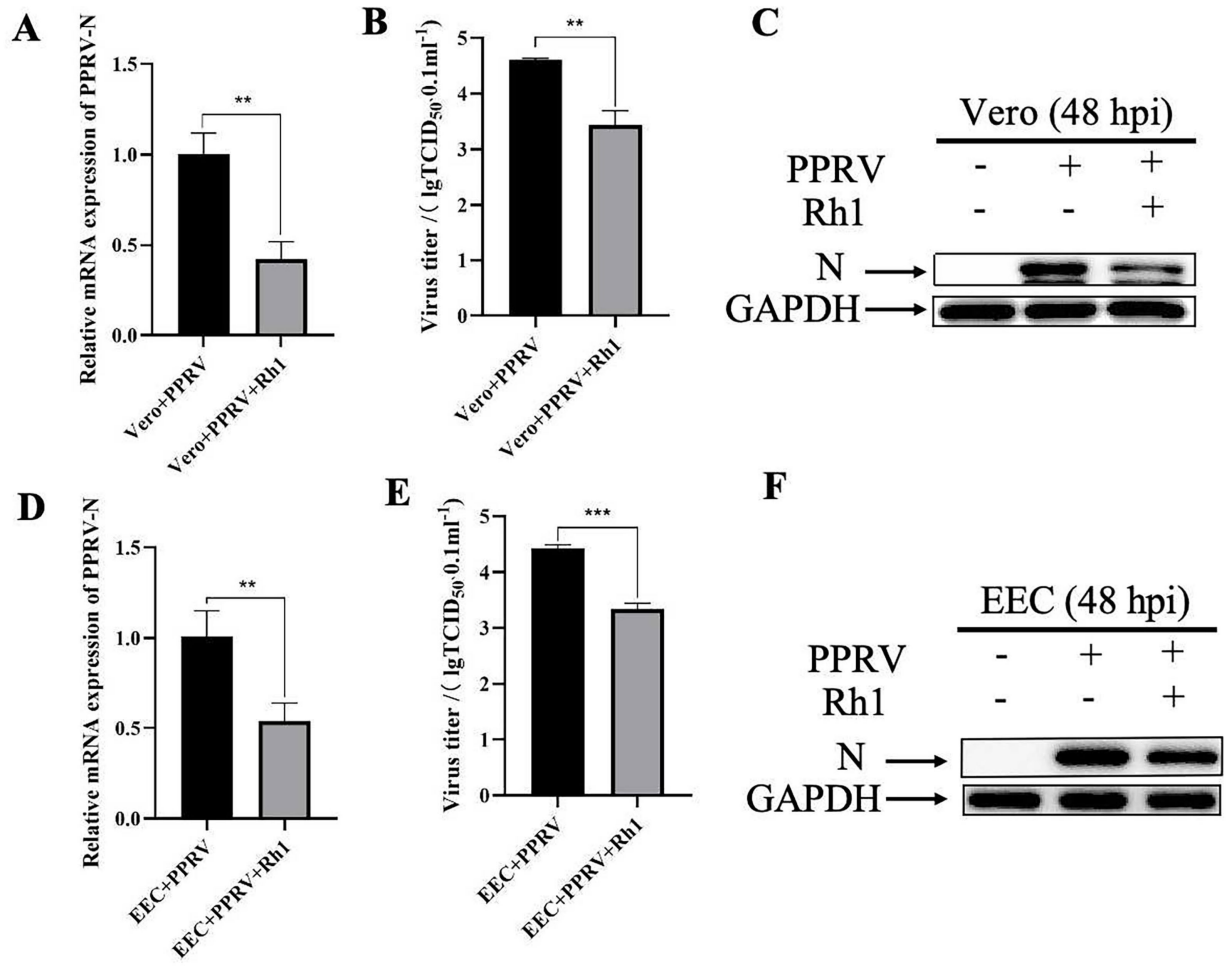
Ginsenoside Rh1 (50 μM) significantly inhibits PPRV replication both in Vero and EEC cells. **(A)** PPRV mRNA levels in 50 μM Ginsenoside Rh1-treated, PPRV-infected Vero cells (MOI = 1, 48 hpi) were measured by qPCR. The data show the means ± SD; *n* = 3; **p < 0.01. **(B)** Control and (50 μM) Ginsenoside Rh1 pre-treated Vero cells were infected with PPRV (MOI = 1), and virus titers were measured by TCID_50_ (48 hpi). The data show the mean ± SD; *n* = 3; **p < 0.01. **(C)** Western blotting analysis of PPRV N protein in PPRV-infected wild-type and Ginsenoside Rh1-treated cells (MOI = 1, 48 hpi). GAPDH was used as a loading control. **(D)** PPRV mRNA levels in 50 μμM Ginsenoside Rh1-treated, PPRV-infected EEC cells (MOI = 1, 48 hpi). The data show the means ± SD; *n* = 3; **p < 0.01. **(E)** Control and (50 μM) Ginsenoside Rh1 pre-treated EEC cells were infected with PPRV (MOI = 1), and virus titers were measured by TCID_50_ (48 hpi). The data show the mean ± SD; *n* = 3; ***p < 0.001. **(F)** PPRV N protein level in infected wild-type and Ginsenoside Rh1-treated EEC cells (MOI = 1, 48 hpi). GAPDH was used as a loading control.

To investigate the impact of Ginsenoside Rh1 on PPRV infection, Vero cells were exposed to varying doses of Ginsenoside Rh1 for 24 h prior to viral challenge and for 48 h post-infection. At 48 hpi, quantitative real-time PCR analysis of cell lysates revealed a dose-dependent decrease in PPRV mRNA levels. While a significant decline was observed at 10 μM Ginsenoside Rh1, treatment with 50 μM resulted in an approximately 70% reduction in viral mRNA compared to the vehicle (DMEM) control (Figure 1A). This suggests that effective antiviral activity requires a relatively high concentration of Ginsenoside Rh1. Importantly, Ginsenoside Rh1 alone did not affect cell viability (Figure 1B), indicating that its inhibitory effect is not attributable to cytotoxicity. In line with this, viral titers (Figure 1C) and PPRV N protein levels (Figure 1D) also declined in a dose-dependent manner with increasing Ginsenoside Rh1 concentrations. To fully investigate the dynamic regulatory effect of Ginsenoside Rh1 on the PPRV replication cycle, viral replication at different time points post-infection (12 h, 24 h, 48 h, 72 h and 96 h) were measured. The data revealed that Ginsenoside Rh1 inhibits PPRV replication in a time-dependent manner. Inhibition of viral mRNA levels (Figure 1E), viral titers (Figure 1F), and PPRV N protein expression (Figure 1G) were observed as early as 24 h post-infection and became more pronounced at 48 h and 72 h, indicating that Ginsenoside Rh1 exerts a sustained antiviral effect against PPRV. Consistently, pretreatment with 50 μM Ginsenoside Rh1 significantly lowered the viral mRNA level (Figures 2A,D) and infectious virus yield (Figures 2B,E) both in Vero and EEC cells and markedly reduced viral nucleoprotein (N) expression at 48 hpi, as shown by western blotting (Figures 2C,F). Together, these results demonstrate that Ginsenoside Rh1 strongly inhibits PPRV replication *in vitro*.

### Ginsenoside Rh1 inhibits the adsorption, invasion and replication of PPRV

**Figure 3:**
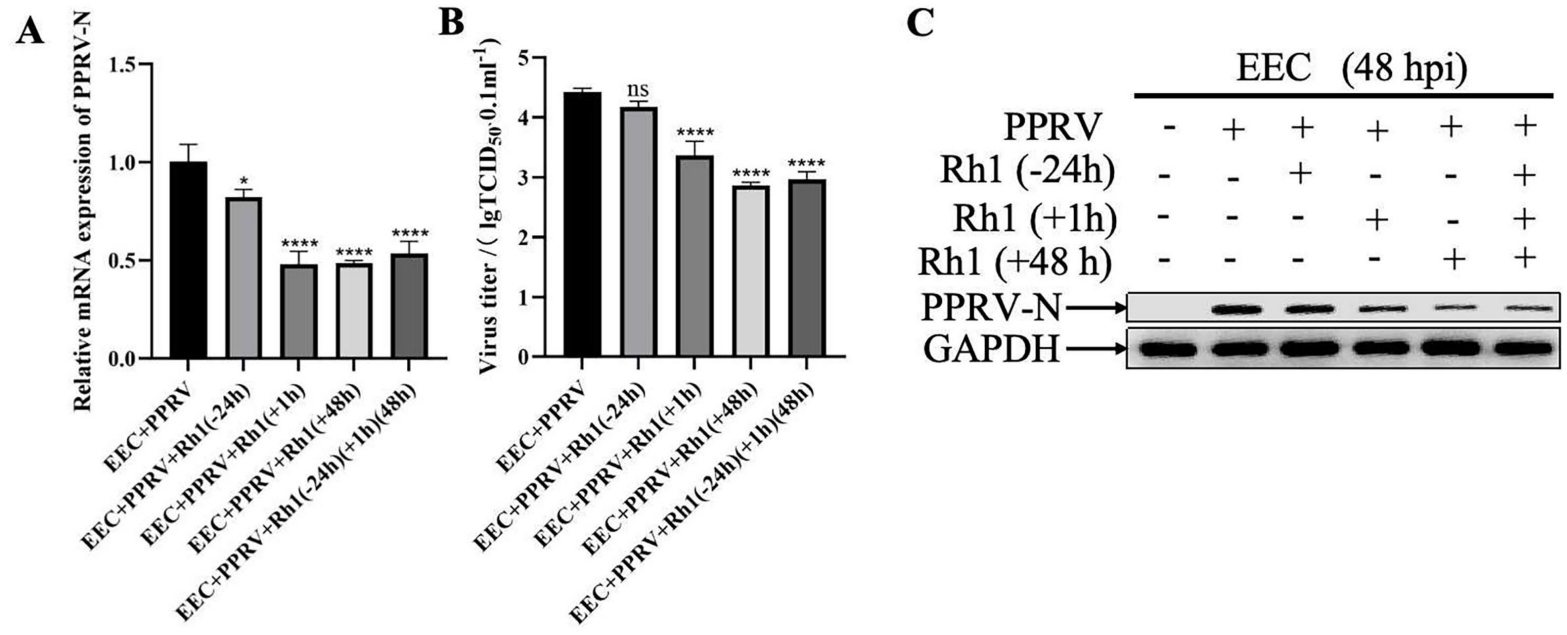
Ginsenoside Rh1 inhibits the adsorption, invasion, and replication of PPRV. **(A)** PPRV mRNA levels in 50 μM ginsenoside Rh1-treated, PPRV-infected EEC cells (MOI = 1, 48 hpi). The data show the means ± SD; *n* = 3; *p < 0.005; ****p < 0.0001. **(B)** Control and (50 M) insenoside Rh1 pre-treated EEC cells were infected with PPRV (MOI = 1), and virus titers were measured by TCID_50_ (48 hpi). The data show the mean ± SD; *n* = 3; ****p < 0.0001; ns, no significance. **(C)** Western blotting analysis of PPRV N protein in PPRV-infected wild-type and Ginsenoside Rh1-treated cells (MOI = 1, 48 hpi). GAPDH was used as a loading control.

We next determined on which stage in PPRV life cycle Ginsenoside Rh1 acts. To this end, EEC cells were either pretreated with 50 μM Ginsenoside Rh1 for 24 h before infection, 1 h during invasion, 48 h post infection, or both before and after infection, respectively. At 48 hpi, both viral RNA levels (Figure 3A) and infectious virus titers (Figure 3B) were significantly reduced in Ginsenoside Rh1-treated cells compared to the control group, as measured by RT-qPCR and TCID_50_ assay. Consistent with this, PPRV N protein levels of Ginsenoside Rh1-treated cells were markedly reduced, as shown by western blotting (Figure 3C). The results indicated that Ginsenoside Rh1 treatment suppressed PPRV replication regardless of timing, but the inhibitory effect was more pronounced when applied post-infection. Collectively, these findings demonstrate that Ginsenoside Rh1 effectively inhibits the attachment, penetration and replication stages of PPRV in vitro.

### Ginsenoside Rh1 promotes PPRV-induced interferon responses and inhibits inflammatory responses

**Figure 4:**
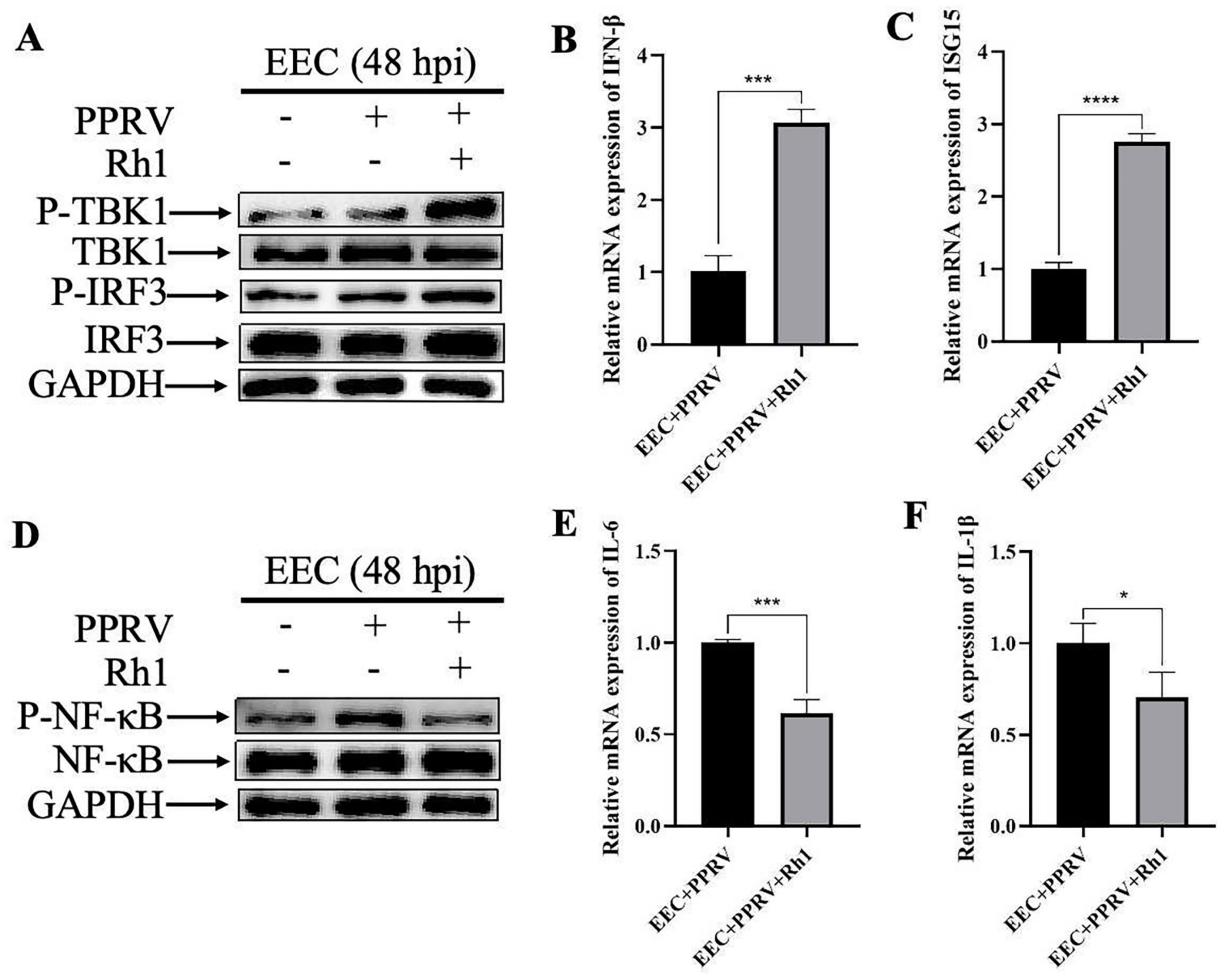
Ginsenoside Rh1 promotes PPRV-induced interferon responses and inhibits inflammatory responses. **(A)** Western blotting analysis of P-TBK1, TBK1, P-IRF3, and IRF3 protein in PPRV-infected wild-type and Ginsenoside Rh1-treated EEC cells (MOI = 1, 48 hpi). GAPDH was used as a loading control. **(B)** IFN-*β* mRNA levels in 50 μM Ginsenoside Rh1-treated or not, PPRV-infected EEC cells (MOI = 1, 48 hpi). The data show the means ± SD; *n* = 3; ***p < 0.001. **(C)** ISG15 mRNA levels in 50 μM Ginsenoside Rh1-treated or not, PPRV-infected EEC cells (MOI = 1, 48 hpi). The data show the means ± SD; *n* = 3; ****p < 0.0001. **(D)** Western blotting analysis of P-NF-κB and NF-κB protein in PPRV-infected wild-type and Ginsenoside Rh1-treated EEC cells (MOI = 1, 48 hpi). GAPDH was used as a loading control. **(E)** IL-6 mRNA levels in 50 μμM Ginsenoside Rh1-treated or not, PPRV-infected EEC cells (MOI = 1, 48 hpi). The data show the means ± SD; *n* = 3; ***p < 0.001. **(F)** IL-1β mRNA levels in 50 μM Ginsenoside Rh1-treated or not, PPRV-infected EEC cells (MOI = 1, 48 hpi). The data show the means ± SD; *n* = 3; *p < 0.005.

To investigate the mechanism by which Ginsenoside Rh1 exerts its anti-PPRV activity, we assessed its impact on innate immune signaling in PPRV-infected EEC cells. Our results showed that Ginsenoside Rh1 potentiated the activation of the TBK1-IRF3 signaling axis triggered by PPRV infection in EEC cells (Figure 4A), leading to a significant upregulation of downstream antiviral effector molecules, IFN-*β* (Figure 4B) and ISG15 (Figure 4C). Conversely, Ginsenoside Rh1 simultaneously suppressed the PPRV-induced activation of the NF-κB pathway in EEC cells (Figure 4D) and markedly inhibited the expression of pro-inflammatory cytokines IL-6 (Figure 4E) and IL-1β (Figure 4F). Taken together, these results indicate that Ginsenoside Rh1 exerts its anti-PPRV activity through a coordinated immunomodulatory strategy.

### Ginsenoside Rh1 inhibits PPRV-mediated autophagy

**Figure 5:**
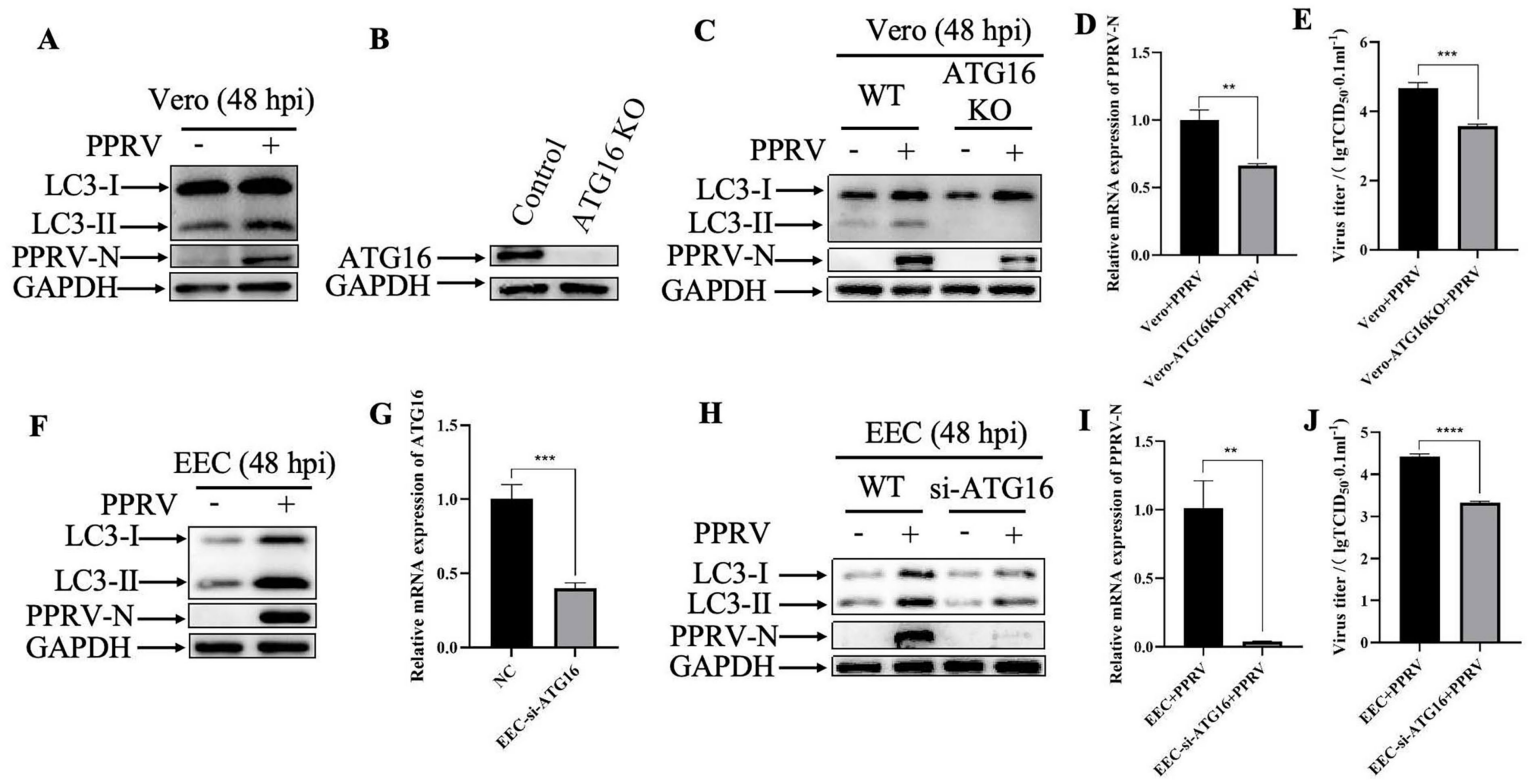
PPRV activates autophagy to promote its replication. **(A)** Western blotting analysis of PPRV N protein and LC3 in PPRV-infected wild-type Vero cells (MOI = 1, 48 hpi). **(B)** ATG16 silencing efficiency were verified by western boltting. GAPDH was used as a loading control. **(C)** Western blotting analysis of PPRV N protein and LC3 in wild-type and ATG16 KO Vero cells infected with or without PPRV infection (MOI = 1, 48 hpi). **(D)** PPRV mRNA levels in PPRV-infected wild-type and ATG16 KO Vero cells (MOI = 1, 48 hpi) were measured by qPCR. The data show the mean ± SD; *n* = 3; **p < 0.01. **(E)** Wild-type and ATG16 KO Vero cells were infected with PPRV (MOI = 1), and virus titers were measured by TCID_50_ (48 hpi). The data show the mean ± SD; *n* = 3; ***p < 0.001. **(F)** Western blotting analysis of PPRV N protein and LC3 in PPRV-infected wild-type EEC cells (MOI = 1, 48 hpi). **(G)** ATG16 mRNA levels in EEC cells transfected with ATG16-siRNA or scrambled siRNA were measured by qPCR. The data show the mean ± SD; n = 3; ***p < 0.001. **(H)** Western blotting analysis of PPRV N protein and LC3 in wild-type and ATG16 KD EEC cells infected with or without PPRV infection (MOI = 1, 48 hpi). **(I)** PPRV mRNA levels in PPRV-infected wild-type and ATG16 KD EEC cells (MOI = 1, 48 hpi) were measured by qPCR. The data show the mean ± SD; *n* = 3; **p < 0.01. **(J)** Wild-type and ATG16 KD EEC cells were infected with PPRV (MOI = 1), and virus titers were measured by TCID_50_ (48 hpi). The data show the mean ± SD; *n* = 3; ****p < 0.0001.

Autophagy is an evolutionarily conserved intracellular degradation process essential for the maintenance of cellular homeostasis through catabolic lysis of otherwise detrimental cytosolic components like viruses. In the attempt to determine the role of autophagy in the antiviral mechanism of Ginsenoside Rh1, cells were infected with PPRV, and western blotting was performed to determine the conversion of LC3-I to LC3-II, which is currently regarded as an accurate indicator of autophagic activity. As shown in Figures 5A,F, compared with control cells, the band intensity of LC3-II in PPRV-infected Vero and EEC cells were dramatically increased at 48 hpi, indicating that PPRV could activate autophagy in Vero and EEC cells. To further confirm the role of auphagy in PPRV replication, ATG16-deficient cells were constructed (Figures 5B,G). The results showed that the deficiency of ATG16 significantly inhibited the autophagy mediated by PPRV infection (Figures 5C,H). In particular, knockout (KO) of ATG16 completely abolished the autophagy flux. Meanwhile, loss of ATG16 dramatically decreased the expression of PPRV structural protein N (Figures 5C,H), viral mRNA levels (Figures 5D,I) and viral titers (Figures 5E,J), when compared to the PPRV-infected wild-type cells. Taken together, these findings suggest that PPRV exploits autophagy to facilitate its replication.

**Figure 6:**
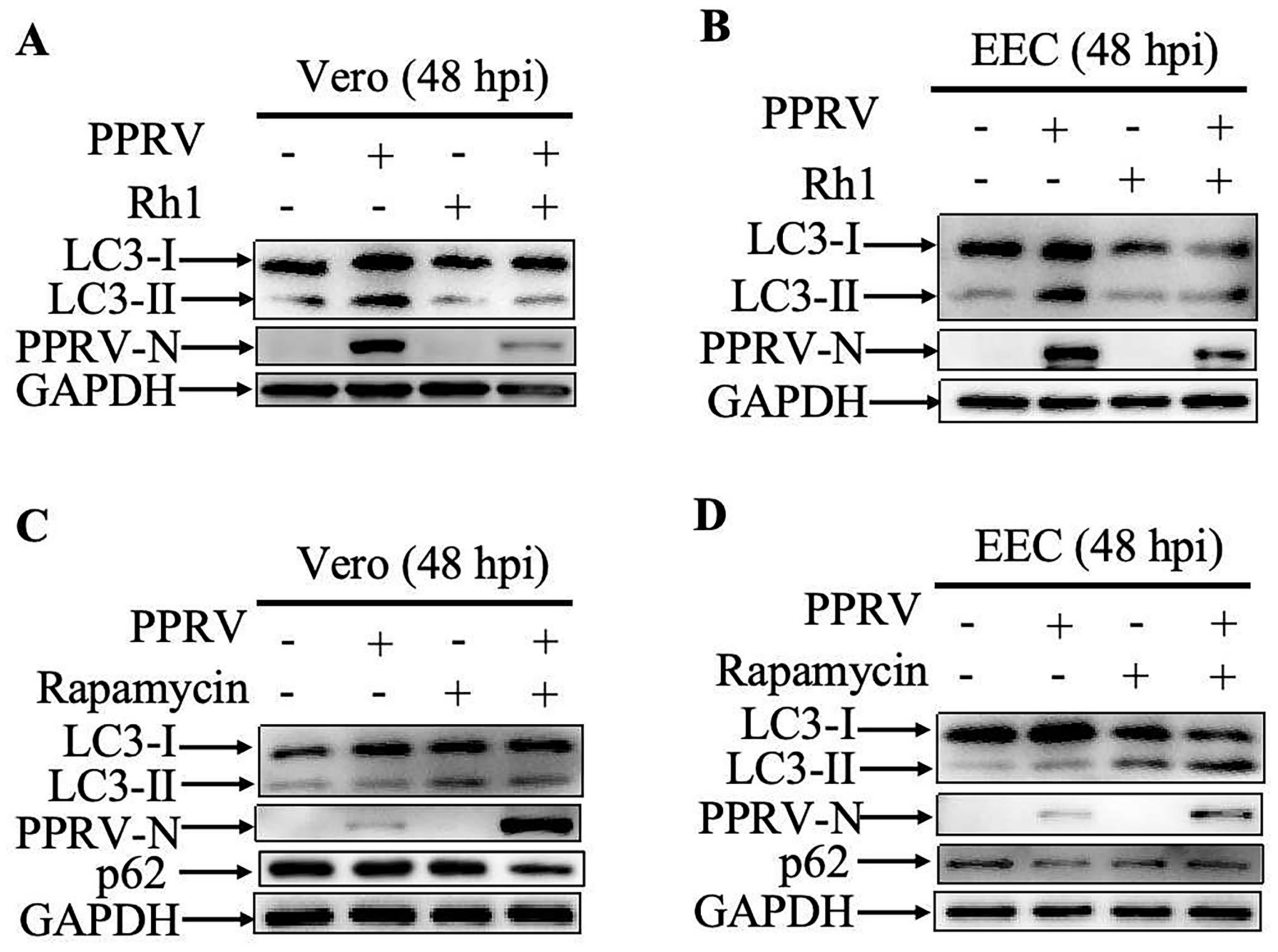
Ginsenoside Rh1 inhibits PPRV-induced autophagy. **(A)** Western blotting analysis of PPRV N protein and LC3 in PPRV-infected, ginsenoside Rh1-treated (50 μM) and untreated wild-type Vero cells (MOI = 1, 48 hpi). **(B)** Western blotting analysis of PPRV N protein and LC3 in PPRV-infected, ginsenoside Rh1-treated (50 μM) and untreated wild-type EEC cells (MOI = 1, 48 hpi). **(C)** Western blotting analysis of PPRV N protein, p62, and LC3 in PPRV-infected, rapamycin-treated (20 μM) and untreated wild-type Vero cells (MOI = 1, 48 hpi). **(D)** Western blotting analysis of PPRV N protein, p62, and LC3 in PPRV-infected, rapamycin-treated (20 μM) and untreated wild-type EEC cells (MOI = 1, 48 hpi).

To further validate the impact of Ginsenoside Rh1 on PPRV-induced autophagy, cells were infected with PPRV at MOI of 1 for 48 h with the treatment of 50 μM Ginsenoside Rh1. Our results showed that treatment with Ginsenoside Rh1 alone had no effect on cellular basal autophagy, however, Ginsenoside Rh1 markedly inhibited the autophagy process triggered by PPRV infection both in Vero and EEC cells (Figures 6A,B). What’s more, activation of autophagy with rapamycin (20 μM) significantly increased autophagy flux and PPRV N protein levels in Vero and EEC cells (Figures 6C,D), confirming that autophagy inhibition directly causes the antiviral effect. In summary, these findings imply that the anti-PPRV activity of Ginsenoside Rh1 is attributable to its ability to inhibit PPRV-induced autophagy.

### Inhibition of autophagy with Ginsenoside Rh1 promotes PPRV-dependent interferon responses and inhibits inflammatory responses

**Figure 7:**
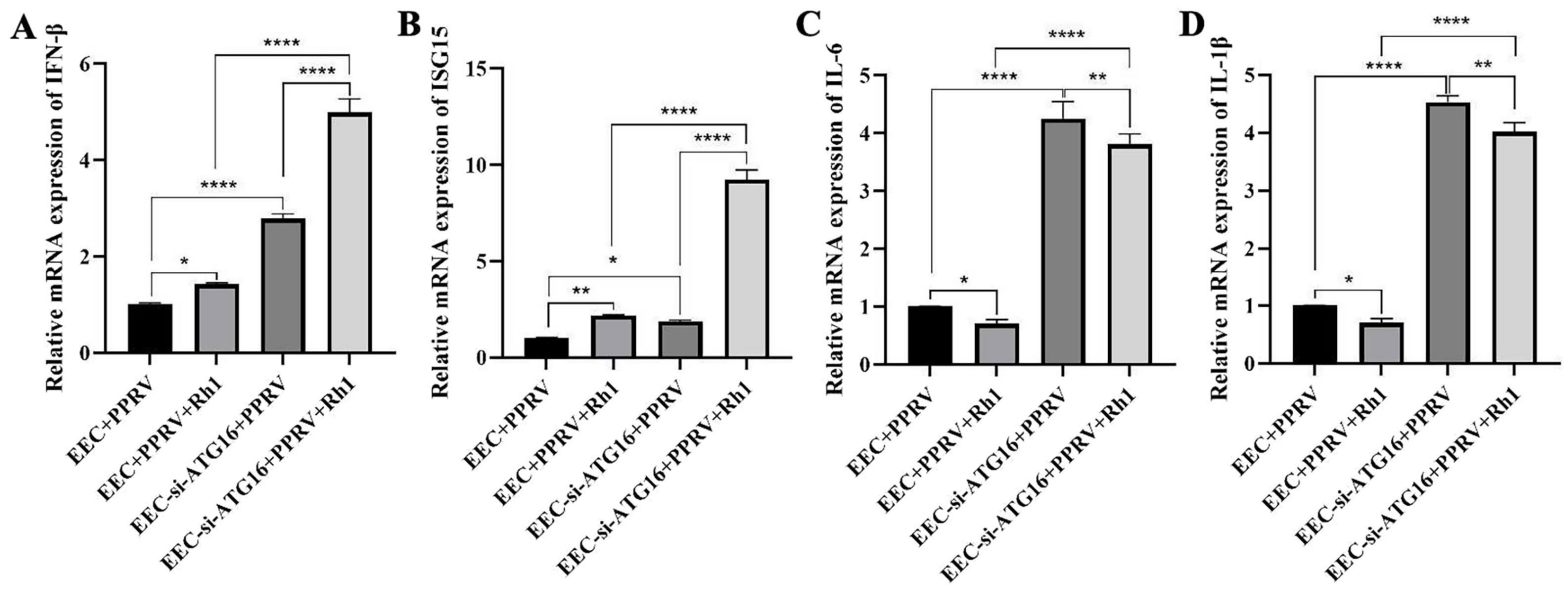
Ginsenoside Rh1 suppresses PPRV-induced auotophagy to promote interferon responses and inhibit inflammatory responses. **(A)** IFN-β mRNA levels in 50 μM Ginsenoside Rh1-treated or not, PPRV-infected wild-type and ATG16 KD EEC cells (MOI = 1, 48 hpi). The data show the means ± SD; *n* = 3; *p < 0.005; ****p < 0.0001. **(B)** ISG15 mRNA levels in 50 M Ginsenoside Rh1-treated or not, PPRV-infected wild-type and ATG16 KD EEC cells (MOI = 1, 48 hpi). The data show the means ± SD; *n* = 3; *p < 0.005; **p < 0.01; ****p < 0.0001. **(C)** IL-6 mRNA levels in 50 M Ginsenoside Rh1-treated or not, PPRV-infected wild-type and ATG16 KD EEC cells (MOI = 1, 48 hpi). The data show the means ± SD; *n* = 3; *p < 0.005; **p < 0.01; ****p < 0.0001. **(D)** IL-1β mRNA levels in 50 M Ginsenoside Rh1-treated or not, PPRV-infected wild-type and ATG16 KD EEC cells (MOI = 1, 48 hpi). The data show the means ± SD; *n* = 3; *p < 0.005; **p < 0.01; ****p < 0.0001.

As PPRV make use of autophagy to promote its replication, abolish of autophagy by ATG16 knockout would lead to a lower viral yield than that of ATG16 knockdown cells, which only partly blocked autophagy. However, according to our observations, PPRV N protein levels (Figure 5C), PPRV mRNA levels (Figure 5D) and virus titers (Figure 5E) in ATG16 KO Vero cells were all higher than those in ATG16 KD EEC cells (Figures 5H–J). Considering Vero cells have a genetic defect in interferon production, we thus speculated that autophagy may have an effect on PPRV-triggered interferon production. To test this hypothesis, we measured the interferon levels both in wild-type and ATG16 KD EEC cells. As shown, compared with PPRV-infected wild-type EEC cells, the mRNA levels of IFN-*β* (Figure 7A) and ISG15 (Figure 7B) were significantly increased in ATG16 deficitive cells. What’s more, the promotion effect of Ginsenoside Rh1 on IFN-β and ISG15 production in PPRV-infected ATG16 KD cells were stronger than that in PPRV-infected wild-type EEC cells (Figures 7A,B), indicating that Ginsenoside Rh1 exerts its anti-PPRV activity by enhancing PPRV-mediated interferon responses through autophagy inhibition. Defectiveness of ATG16 increased pro-inflammatory cytokines IL-6 (Figure 7C) and IL-1β (Figure 7D) production as well. However, Ginsenoside Rh1 had an opposite effect on these pro-inflammatory cytokines production (Figures 7C,D) when compared with its promoting effect on interferon responses, and the inhibition effect of Ginsenoside Rh1 on IL-6 and IL-1β production in PPRV-infected ATG16 KD cells were stronger than that in PPRV-infected wild-type EEC cells. Taken together, these findings suggest that the antiviral activity of Ginsenoside Rh1 is due to its inhibitory effect on PPRV-induced autophagy resulted in potent promotion of interferon responses and inhibition of inflammatory responses.

## Discussion

PPRV poses a severe threat to global small ruminant populations due to its high contagion and mortality rates (1), having already incurred substantial economic losses in endemic regions (50). Currently, vaccination represents the primary strategy for controlling PPRV transmission. Live attenuated vaccines are widely deployed and serve as cornerstone tools in international eradication efforts. Nevertheless, their effectiveness is constrained by thermolability—a characteristic shared among paramyxoviruses—necessitating stringent cold-chain logistics, especially in warmer climates, which substantially escalates distribution costs (51). These limitations underscore the pressing demand for the development of antiviral therapeutics capable of complementing or supplementing vaccination. To date, however, no antiviral agents specifically targeting PPRV have been reported, highlighting a critical gap in the current management.

Ginseng is a medicinal herb sourced from the roots of several species, most notably *Panax ginseng* (Korean/Asian ginseng), *Panax quinquefolius* (American ginseng), and *Eleutherococcus senticosus* (Siberian ginseng) (52). It has been widely employed in traditional medicine systems for its purported benefits in modulating immune responses and enhancing physical performance (31). Ginseng is predominantly administered orally. Ginsenosides are considered the major components of ginseng, following ingestion, intestinal microbial flora metabolize major ginsenosides into bioactive derivatives (27, 32). Fermented ginseng preparations have demonstrated a range of physiological effects, such as antioxidant and antibacterial properties (28), mitigation of dextran sulfate sodium-induced colitis (29), and antidiabetic activity (30). Several ginsenosides have demonstrated antiviral properties across different viral models. 20(R)-ginsenoside Rh2 suppressed gamma herpesvirus replication in murine and human systems (53), while 20(S)-protopanaxtriol exhibited potent activity against coxsackievirus B3 (CVB3) *in vitro* (54). Notably, ginsenosides Re, Rf, and Rg2 conferred protection against rhinovirus 3 and coxsackievirus infections. Additionally, Rg2 has shown significant anti-Enterovirus 71 (EV71) activity (55). Ginsenoside Rg3 inhibited Hepatitis C virus (HCV) -induced persistent infection (56). Furthermore, Rb2 reduced viral titers and provided protection against rotavirus and Sendai virus infections in mice (57, 58). Ginsenoside Rb2 and Rb3 demonstrate therapeutic potential against Pestivirus infections, including bovine viral diarrhea virus (BVDV) and classical swine fever virus (CSFV) in vitro (59). Fermented ginseng extracts inhibited a broad-spectrum of influenza viruses (32). The findings by Kang et al. (23) established Ginsenoside Rb1 as an immunostimulatory agent with antiviral efficacy against EV71 in both cellular and animal models. In line with this, our study demonstrates that Ginsenoside Rh1 also exerts significant inhibitory effects on PPRV replication in vitro. This parallel suggests that the antiviral properties of certain ginsenosides may not be virus-specific but rather represent a broader, host-directed mechanism.

The control of pathogenic microbes involves a coordinated immune response, wherein innate immunity acts as the primary barrier, followed by pathogen-specific clearance mediated by the adaptive system (60, 61). A cornerstone of innate defense is the IFN response, which rapidly induces the expression of key immune mediators such as cytokines and chemokines, forming the essential early layers of host protection. Ginseng is widely used as herbal tonic due to its regulation of immune function (21). Studies have shown that Ginsenoside Rb1 can enhance both cellular and humoral immune responses *in vivo* and potentiate IFN signaling. Knockdown of IFN-*β* abolished the antiviral effect of Ginsenoside Rb1. Furthermore, Rb1 treatment has been reported to upregulate IFN-*α*, IFN-β, and the IFN-induced protein MxA in specific viral infection models (55, 62, 63). While Ginsenoside Rh1 is recognized for its anti-inflammatory effects on ameliorating asthma in mice by restoring Th1/Th2 cytokine balance (31, 64). Studies have shown that ginseng extracts attenuate the release of pro-inflammatory cytokines IL-6 and IL-8 and stimulate interferon (IFN)-β expression in mice challenged with influenza virus (65, 66). Tao Yu et al. showed that Ginsenoside molecules markedly suppress the expression of cytokines, such as TNF-α and IL-1 (67). Our results showed that Ginsenoside Rh1 significantly promotes the activation of TBK1-IRF3 signaling axis and enhances the expression of type I interferon and its downstream antiviral effector, ISG15, in response to PPRV infection. The potentiation of the TBK1-IRF3 axis by Ginsenoside Rh1 suggests it may act to counteract viral immune evasion strategies. PPRV, like other morbilliviruses, encodes multiple proteins that antagonize the IFN response. The V protein targets STAT1/2 and JAK1 (68–70), while the N and P proteins inhibit IFN signaling by blocking STAT1 nuclear translocation or interacting with IRF3 (71). It is therefore conceivable that Rh1 could be relieving a PPRV-imposed blockade at the level of TBK1 or its upstream activators, such as the RNA sensors RIG-I and MDA5. Interestingly, Zhang et al. (15) demonstrated that PPRV infection upregulates STING, which can activate ATF6-mediated autophagy. The relationship between Rh1, the MDA5-STING axis, and the subsequent activation of TBK1 represents a compelling avenue for future research. In alignment with its known anti-inflammatory properties, we concurrently observed that Ginsenoside Rh1 suppresses the activation of NF-κB and the expression of PPRV-induced pro-inflammatory cytokines. Given viral replication itself is a potent trigger for NF-κB activation and cytokine production, the reduced inflammation observed could be, at least in part, a consequence of the lower viral load resulting from Ginsenoside Rh1’s antiviral activity. We speculate that Ginsenoside Rh1 may exert a dual mechanism: directly suppressing the NF-κB pathway while also limiting the viral trigger that activates it. The definitive mechanistic clarification requires further study. Regardless of the precise mechanism, the concurrent suppression of viral replication and inflammation by Rh1 suggests it may offer therapeutic advantages by targeting both viral and host factors contributing to disease pathogenesis. Our findings suggest that Ginsenoside Rh1 selectively potentiating the antiviral interferon response while attenuating detrimental hyperinflammation. The importance of controlling hyperinflammatory responses is underscored by recent systematic reviews documenting rare but severe immune-related complications following vaccination, including hemophagocytic lymphohistiocytosis (72), Sweet syndrome (73), and Vogt-Koyanagi-Harada disease (74). These conditions are driven by dysregulated cytokine storms. The ability of Ginsenoside Rh1 to suppress NF-κB-driven pro-inflammatory cytokines, while simultaneously potentiating antiviral interferon signaling, suggests it could offer a balanced immunomodulatory approach that may be relevant for managing not only viral infections but also vaccine-associated hyperinflammatory conditions. This coordinated immunomodulation positions Ginsenoside Rh1 as a promising candidate for developing balanced therapeutic strategies against viral diseases like those caused by PPRV, where effective control requires both robust antiviral defense and mitigated immunopathology.

Autophagy is an evolutionarily conserved lysosomal degradation pathway essential for maintaining cellular homeostasis and eliminating intracellular pathogens (75). However, numerous viruses (76), including MV (77), HCV (78), foot-and-mouth disease virus (FMDV) (79), influenza A virus (IAV) (72) and dengue virus (DENV) (80), have evolved mechanisms to manipulate autophagy to evade immune clearance and promote their own replication. Similar to MV, PPRV, a morbillivirus closely related to MV, has been shown to hijack the autophagic machinery to facilitate its replication (48, 81). Our study validated that PPRV infection activates autophagy and inhibition of autophagy significantly decreased the viral replication. However, we acknowledge a limitation in our study regarding the characterization of autophagy. Our conclusions on the inhibitory effect of Ginsenoside Rh1 on PPRV-induced autophagy are primarily based on steady-state measurements of LC3-II conversion and ATG16 expression. While these are well-established markers for autophagosome formation, they do not fully capture the dynamic nature of autophagic flux. Without flux analysis, we cannot definitively distinguish between reduced autophagosome formation and enhanced autophagosome clearance, future studies should employ a comprehensive set of assays to rigorously assess autophagic flux.

Autophagy serves not only as a fundamental cellular defense mechanism that eliminates microbial pathogens through autophagosomal degradation but also plays a pivotal role in initiating both innate and adaptive immune responses (45, 82, 83). The interplay between autophagy and innate immunity is complex. It has been reported that ATG13 exerts antiviral activity against PPRV by enhancing IFN production and depletion of ATG13 markedly diminished the capacity of RIG-I to activate IFN responses (84). Conversely, we found that genetic inhibition of autophagy by silencing ATG16 upregulated both antiviral mediators (IFN-*β* and ISG15) and pro-inflammatory cytokines (IL-1β and IL-6). This apparent paradox highlights the multifaceted roles of ATG proteins, which can function both as part of the core autophagy machinery and as signaling adaptors in immune pathways. Furthermore, our study demonstrated that Ginsenoside Rh1 inhibited PPRV-induced autophagy to suppress PPRV replication. Strikingly, treatment with Ginsenoside Rh1 recapitulated the autophagy-inhibitory effect but elicited a more refined immunomodulatory outcome: it enhanced the expression of IFN-β and ISG15 while simultaneously suppressing the production of IL-1β and IL-6. Recent work by Lv et al. (85) has elegantly demonstrated that M2 macrophage-derived extracellular vesicles can enhance the function of group 2 innate lymphoid cells (ILC2s) in allergic inflammation, mediated by specific long non-coding RNAs. Similarly, the work of Liu et al. (86) provides a powerful example of therapeutic immunomodulation, representing a direct precedent for using an immunomodulatory agent to counteract a pathogen- or therapy-induced immunosuppressive feedback loop. This observed dual immunomodulatory effect of Ginsenoside Rh1: enhancing antiviral interferon signaling while attenuating pro-inflammatory cytokine production—suggests a sophisticated and potentially therapeutically superior mechanism of action. Therefore, Ginsenoside Rh1 emerges not merely as an autophagy inhibitor, but as a precision immunomodulator that recalibrates the host response to viral infection.

While our *in vitro* findings demonstrate that Ginsenoside Rh1 effectively inhibits PPRV replication at a concentration of 50 μM, it is crucial to consider the pharmacological feasibility and translational potential of this observation. Previous pharmacokinetic studies have shown that ginsenosides, including Rh1, can be absorbed and detected systemically following oral administration of ginseng extracts or fermented products, although their absolute bioavailability is often limited due to poor membrane permeability and extensive metabolism (31, 32). The concentration of 50 μM used in our study may be higher than typical steady-state plasma levels achieved through conventional oral dosing. However, several factors could bridge this gap. First, the potent immunomodulatory and autophagy-inhibitory effects of Rh1 might amplify its antiviral activity *in vivo*, potentially allowing for efficacy at lower, physiologically achievable concentrations. Second, tissue distribution studies suggest that certain ginsenosides can accumulate in target organs such as the lungs and lymphoid tissues—sites of active PPRV replication—potentially reaching local concentrations higher than those in plasma. Third, alternative delivery strategies, such as intranasal or aerosolized administration, could be explored to directly target the respiratory epithelium, the primary portal of PPRV entry, thereby enhancing local bioavailability while minimizing systemic exposure. Future studies employing appropriate animal models of PPRV infection are therefore essential to rigorously evaluate the in vivo pharmacokinetics, tissue distribution, and therapeutic efficacy of Ginsenoside Rh1. Such investigations will be critical to determine whether the antiviral and immunomodulatory properties we have identified can be translated into a practical and effective intervention for controlling PPRV in the field.

In conclusion, we demonstrated for the first time that Ginsenoside Rh1 effectively inhibits PPRV replication in vitro. Evaluation of the mechanism of action of Ginsenoside Rh1 against PPRV revealed that the antiviral activity is due to the blockage of PPRV mediated autophagy, thus leading to stimulation of interferon responses and inhibition of inflammatory responses, highlighting that modulation of autophagy represents an attractive approach to counteract viruses infection. Since Ginsenoside Rh1 is a clinically wildly used herbal medicine, our data might provide a new therapeutic option for the cure and possibly also the prevention of viral diseases in humans like measles.

## Data Availability

The raw data supporting the conclusions of this article will be made available by the authors, without undue reservation.
